# Expressive Suppression Within Task-Oriented Dyads: The Moderating Role of Power

**DOI:** 10.5964/ejop.v16i4.1947

**Published:** 2020-11-27

**Authors:** Stefania Balzarotti, Stefano Cesana, Federica Biassoni, Maria R. Ciceri

**Affiliations:** a Department of Psychology, Catholic University of the Sacred Heart, Milan, Italy; University of Belgrade, Belgrade, Serbia

**Keywords:** expressive suppression, nonverbal behavior, emotional experience, power

## Abstract

Although research has so far consistently revealed that using suppression to regulate emotions has adverse personal and social effects, it has been argued that suppression may be less detrimental within non-close relationships. In the present work, we examined the effects of experimentally induced suppression on expressive behavior, emotional experience, and social outcomes within task-oriented interactions between individuals randomly assigned to high/low vs. equal power positions. Eighty-eight participants were randomly paired with a partner of the same gender (forty-four dyads). After being randomly assigned to one of four experimental conditions created to manipulate suppression use and power, each dyad was asked to complete two problem-solving tasks. The results showed that the participants who were assigned to the subordinate (low-power) role and who used suppression to regulate their emotions reported more negative emotional experience than did individuals assigned to equal-power roles, as well as more inauthenticity and diminished feelings of rapport compared to subordinates who freely expressed their feelings. Moreover, we found that the use of suppression also influenced participants assigned to the manager (high-power) role, as they exhibited less positive behavior, reported less positive experience and lower feelings of rapport when interacting with a partner asked to suppress. When individuals were assigned to equal power roles, the participants instructed to use suppression reported lower levels of positive emotions than did their partners as well as higher feelings of inauthenticity compared to uninstructed participants. Overall, these findings seem to suggest that suppression may impair task-oriented interactions between high/low power individuals more than interactions between individuals sharing equal power.

A common strategy people use to regulate their emotions is expressive suppression, which refers to the attempt to hide and avoid the outward display of positive and negative feelings (Butler et al., 2003; Gross, 1998). Compared to other strategies, suppression has been considered as a more interpersonal form of emotion regulation, since it directly targets outer expressive behavior which is visible to others (Impett et al., 2012). This consideration has led researchers to study the implications that using this strategy may have for the individual’s social functioning (Butler et al., 2003; English & John, 2013; English, John, & Gross, 2013; English, John, Srivastava, & Gross, 2012; Gross & John, 2003; Impett et al., 2012; Srivastava, Tamir, McGonigal, John, & Gross, 2009; Velotti et al., 2016). Overall, existing evidence suggests that hiding feelings has a number of negative side effects on relationships, leading individuals to experience higher inauthenticity, less social support, and lower social satisfaction (Gross & John, 2003). Similarly, partners of habitual suppressors report lower relationship closeness and interpersonal warmth (English et al., 2012; Srivastava et al., 2009). Most research has adopted an individual-difference, correlational approach; however, a few studies using experimental manipulation have also found harmful effects such as lower perceived responsiveness and less willingness to form a friendship (Butler et al., 2003).

Although these studies have deepened our understanding of the effects that concealing feelings may have on social functioning, research has so far mainly focused on close relationships (English & John 2013; English et al., 2012; Gross & John, 2003; Impett et al., 2014; Le & Impett, 2013; Srivastava et al., 2009; Velotti et al., 2016) and it is thus still unclear whether these detrimental social outcomes generalize to other types of social contexts. People may choose to inhibit their emotional reactions when dealing with various types of social partners, including strangers or casual acquaintances, and it has been proposed that suppression use may not impair non-close relationships or interactions that do not involve intimacy and emotional disclosure (English et al., 2013). Also, other prevalent features of social relationships such as power differences may moderate suppression effects.

In attempting to address these gaps, the current study adds to the existing literature in two ways. First, we examined the social outcomes of instructed suppression within task-focused dyads, that is, interactions that do not involve intimacy and emotional sharing. Second, we examine whether power differences moderate these social outcomes by using randomly assigned positions of power. The study takes into account the consequences for both the regulator and his or her partner in terms of nonverbal expressive behavior, emotional experience, and social outcomes (e.g., mutual liking, and feelings of rapport).

## Expressive Suppression and Its Consequences for the Individual’s Social Functioning

Within the theoretical framework of the process model of emotion regulation (Gross, 2001, 2015), expressive suppression has been conceptualized as belonging to the so-called category of ‘response-focused’ strategies. Because they intervene late in the emotion generation process, these strategies are thought to be more resource demanding and generally less effective at modulating emotion compared to other forms of regulation (for a review, see Gross, 2007). Consistent with this theoretical prediction, many studies have shown that the use of suppression effectively reduces the outward display, but not the subjective experience of negative emotion (Gross, 1998). Moreover, suppression has been associated with adverse side effects such as increased physiological arousal (e.g., Gross, 1998), impaired memory (e.g., Richards & Gross, 1999), and negative social effects (e.g., Butler et al., 2003).

Among the detrimental consequences of suppression for the individual’s psychological functioning, much attention has been given to its social implications. Suppression directly targets outward expressive behavior, which is considered to be critical to the regulation of social interactions (e.g., Keltner & Kring, 1998; Van Kleef, 2009). Several correlational studies have investigated the social consequences of habitually using suppression across various domains of social functioning in everyday life; these studies have found that habitual suppression is associated with less social support, less relationship closeness, and lower relationship satisfaction (e.g., English & John, 2013; English et al., 2012; Gross & John, 2003; Srivastava et al., 2009; Velotti et al., 2016). Moreover, habitual suppression has been linked to reduced feelings of subjective authenticity (Gross & John, 2003), which has been found to mediate the association of this regulatory strategy with measures of social functioning (English & John, 2013; Impett et al., 2012).

Comparatively fewer studies have examined the social costs of expressive suppression using an experimental paradigm (i.e., instructing participants to suppress their emotions). While correlational studies have focused on close relationships (e.g., spouses, friends; Gross & John, 2003; Velotti et al., 2016), experimental research has manipulated suppression to test the effects of this strategy on social exchanges among relative strangers to eventually understand its consequences on relationship formation. It has been hypothesized that suppression may disrupt two main processes within face-to-face interactions (Butler et al., 2003). First, it may interfere with interpersonal coordination in the service of accomplishing various joint goals by reducing responsive behavior; second, it may impair partners’ negotiation of interpersonal distance and intimacy by inhibiting emotional expressive cues. This can lead the partners to experience distress, to have difficulties in accomplishing joint tasks, to feel less connected and less willing to form a friendship with the suppressing individual.

Existing evidence supports these predictions. For instance, Butler et al. (2003) asked pairs of previously unacquainted women to discuss their thoughts and feelings after viewing an upsetting film. Unbeknownst to the partner, one person in each dyad was asked to hide her emotional reactions. The results showed that the participants instructed to suppress their emotions exhibited less responsive behavior, higher distraction, and increased blood pressure (i.e., higher stress) during the conversation than did the control participants. Moreover, they reported less positive and more negative emotions about the interaction. Similarly, the partners of suppressors showed higher blood pressure during the conversation than did controls; moreover, they also reported less rapport and less willingness to form a friendship. Using a similar procedure, other studies have found that instructed suppressors were judged as more hostile and withdrawn by their partners compared to controls (Butler, Lee, & Gross, 2007) and that suppression use had contagious interpersonal effects, leading partners of suppressors to exhibit less positive and less negative expressive behavior (Butler, Gross, & Barnard, 2014). Finally, Lebowitz and Dovidio (2015) found that participants instructed to suppress while reading information about the distress and misfortune of a fictitious individual reported less empathic concern.

## Potential Moderators of Social Consequences of Suppression

Thus far, both experimental and correlational studies have consistently revealed that using suppression to regulate emotions leads to adverse social effects. However, it has been argued that these social outcomes may vary according to the type of social context (e.g., Butler et al., 2003; Clark & Finkel, 2005; English et al., 2013).

### Close vs. Non-Close Relationships

Among the characteristics that differentiate individuals’ social relationships, most researchers have focused on closeness, advancing the idea that expressive suppression may especially impair the development and maintenance of close relationships, while having less significant effects on other types of interactions and relationships in which mutual self-disclosure and intimacy are not expected (English et al., 2013). For instance, correlational studies examining the link between habitual suppression and peer-rated social functioning have shown that this strategy is associated with peer reports of reduced interpersonal warmth and closeness to others, whereas it does not significantly impact the sociometric standing (e.g., likability, social status) of the regulator (English et al., 2013; Gross & John, 2003; Srivastava et al., 2009). This result suggests that, although it impairs the formation of close bonds, suppression does not hinder the individual’s chance to make a positive impression on others or to achieve social status (Fatigante et al., 2016).

Along with this line of reasoning, there is evidence that people are less willing to display their emotions and report less liking for partners who do so within exchange (e.g., business) relationships compared to communal relationships (e.g., friends, romantic partners; Clark & Finkel, 2005; Clark & Taraban, 1991). In exchange relationships, individuals expect an immediate reciprocation of benefits (rather than being concerned for the other’s needs), and thus interpersonal distance is seen as normative or desirable (Clark & Mills, 1979).

Nevertheless, experimental studies directly examining the social outcomes of suppression have found that concealing feelings leads to various detrimental consequences (e.g., greater stress, lower feelings of rapport) for both the regulator and his or her partner even in interactions between unacquainted individuals (Butler et al., 2003, 2007), thus suggesting that suppression may impair social interactions more broadly. This research, however, has used tasks that require emotional self-disclosure between the partners, such as talking about one’s own emotions in response to a shared negative event. It is therefore still unclear whether the results may extend to task-oriented dyads (or groups; Gross, Guerrero, & Alberts, 2004) ‒ that is, to situations in which unacquainted individuals are asked to accomplish a joint task that does not entail any emotional content.

Overall, more experimental research is needed to understand the social consequences of suppression in non-close relationships.

### Power Disparity

A second characteristic of human relationships that may moderate the social effects of suppression is power, which has been broadly defined as the influence that an individual exerts over other people through the allocation of resources and punishments (Anderson & Berdahl, 2002; Fiske, 1993; Kelley & Thibaut, 1978; Van Kleef, De Dreu, Pietroni, & Manstead, 2006). Past evidence suggests that power affects a wide range of social behaviors, including emotional expression. For instance, people with low power have been found to talk less (Dovidio, Brown, Heltman, Ellyson, & Keating, 1988), to exhibit more inhibitive nonverbal behavior (Ellyson & Dovidio, 1985), and to engage in more facial actions that inhibit emotional displays such as lip press (Keltner, Young, Heerey, Oemig, & Monarch, 1998), compared to people with high power. By contrast, powerholders tend to express their emotions more (Hecht & LaFrance, 1998) and to pay less attention to others (Fiske, 1993; Goodwin, Operario, & Fiske, 1998; Hall, Carter, & Horgan, 2001; Keltner & Robinson, 1996, 1997).

Two main theories have been formulated about power. First, the approach-inhibition theory of power (Keltner, Gruenfeld, & Anderson, 2003) has posited that having high power activates the behavioral approach system (reflecting motivation to respond to and approach potentially rewarding situations), while having low power activates the inhibition system (which reflects motivation to withdraw from potential threats or punishments). Thus, compared to powerholders, people with low power are expected to be more inhibited in the expression of their opinions in order to avoid conflicts (Anderson & Berdahl, 2002). This theory also predicts that people with low power will experience and express more negative and less positive affect than will people with high power (e.g., Berdahl & Martorana, 2006; Langner & Keltner, 2008).

Second, theorists have more recently posited that power creates social distance (Magee & Smith, 2013; Smith & Trope, 2006), such that individuals within power relationships experience more social distance from each other (i.e., lower closeness) than do people within symmetrically dependent relationships. According to this approach, individuals are motivated to affiliate with each other and have expectations of closeness when their relationship is characterized by equal power and shared control over resources. By contrast, within power relationships, the high- and low-power parties will differ (Lammers, Galinsky, Gordijn, & Otten, 2012): Because power-holders are less dependent on their low-power counterparts to satisfy their goals, they will be less motivated to affiliate and will expect efforts to approach and affiliate from their low-power counterparts; thus, they will experience higher social distance than low-power individuals. In this view, power-holders are expected to be less concerned with and less responsive to others’ mental states, as well as less accurate in recognizing others’ emotions.

Thus far, little research has examined the moderating role of power differences on the affective and social consequences of suppression, and the few available results are mixed. Using an experience sampling design, Catterson et al. (2017) found that, in daily life, individuals were more likely to rely on suppression in situations in which they feel low in social hierarchy; moreover, in such situations, the negative consequences of the use of this regulatory strategy on well-being were reduced (though still significant). Moreover, suppression and social hierarchy were negatively associated. However, these findings were not replicated within a different cultural context (i.e., Poland; Pilch et al., 2018), as in this research no significant interaction between suppression and social hierarchy emerged in the prediction of individuals’ well-being. Finally, in a correlational study examining the consequences of emotion regulation strategies for school directors’ (leaders) and teachers’ (subordinates) work-related outcomes, Kafetsios et al. (2012) found that suppression use in subordinates was related to lower job satisfaction and higher to negative affect, thus indicating that the use of this strategy may have negative affective consequences for low-power individuals.

## The Present Study

The present study examined the effects of instructed suppression on emotional expressive behavior, emotional experience, and social aspects (e.g., status, feelings of rapport, mutual liking) within task-oriented interactions between individuals randomly assigned to high/low vs. equal power positions. While existing research examining the social consequences of expressive suppression has mostly targeted close relationships, the present study (1) focuses on temporary task-oriented dyads (i.e., unacquainted dyads assigned to work on a task; Gross et al., 2004; Meyerson, Weick, & Kramer, 1996) rather than on participants required to share their emotions, and (2) examines the potential moderating effect of power.

Although our experimental task did not include any emotional content, we nonetheless expected that emotions would be generated during the interactions. First, dyads were asked to complete problem-solving tasks, which can generate both positive and negative feelings (e.g., in response to failure or success; e.g., Nummenmaa & Niemi, 2004). Second, conflict and disagreement can easily arise within interactions between temporary dyads, in which members who have never worked together are asked to complete a task over a limited period of time (Gross et al., 2004).

The study adopted a dyadic design, as participants were paired to form dyads. Three variables were manipulated by giving instructions to participants: suppression use (Butler et al., 2003), power (Galinsky, Gruenfeld, & Magee, 2003), and role. In the *Suppression* condition, unbeknownst to the partner, one member of the dyad was asked to conceal the outward display of his or her emotions; in the uninstructed (control) condition, both members of the dyad were free to respond as they typically would. Concerning *Power* and *Role,* in the power disparity condition, participants were randomly assigned to either a manager (high-power) or subordinate (low-power) role, while in the equal power (control) condition, the members of the dyad were instructed to cooperate as peers. For the purpose of simplifying our design, only subordinates received the suppression instructions in the power disparity condition. Whereas suppression and power were manipulated at the dyad level, role was manipulated at the individual level. Thus, each participant was assigned to one of following roles: manager (interacting with either an instructed or uninstructed subordinate), subordinate (either receiving instructions to suppress or acting spontaneously), or cooperator (either receiving instructions to suppress or free to express his or her emotional reactions; either interacting either with an instructed or uninstructed partner).

The study examined multiple outcomes of suppression use. Specifically, we considered (a) expressive behavior by using video-coded data (i.e., positive and negative emotional expressions), (b) self-reported emotions experienced during the interaction, and (c) participants’ ratings of social outcomes (i.e., social status, feelings of inauthenticity during the interaction, feelings of rapport, mutual liking). We limited our sample to same-sex dyads to simplify our design (Butler et al., 2003) and measured trait suppression and dominance ‒ a personality trait associated with power, as people high in dominance have been shown to attain more power in dyadic interactions with strangers (Anderson & Berdahl, 2002).

Drawing from prior experimental studies employing unacquainted dyads (Butler et al., 2003), we hypothesized that the use of suppression would also lead to detrimental affective and social consequences in the context of task-focused dyads. In more detail, concerning the personal consequences of suppression for the regulators:

H1. We expected that suppression would reduce regulators’ positive and negative expressivity. Thus, we hypothesized that participants instructed to suppress would show less positive and less negative emotional behavior compared to (H1a) uninstructed participants as well as (H1b) to regulators’ partners in both the disparity and equal power conditions.

H2. We predicted that participants instructed to suppress would report less positive and more negative emotional experience compared to (H2a) uninstructed participants as well as (H2b) to regulators’ partners in both the power disparity and equal power conditions.

H3. We expected that regulators would report heightened feelings of inauthenticity compared to (H3a) uninstructed participants and (H3b) to their partners.

H4. Finally, concerning the moderating role of power, we expected that suppression would have more harmful consequences for participants assigned to the low-power role than for participants in the equal power condition. Thus, we hypothesized that participants instructed to suppress in the power disparity condition (subordinates) would report less positive and more negative emotional experience (H4a), as well as higher inauthenticity (H4b) compared to participants asked to suppress in the equal power condition (cooperators).

Another group of hypotheses concerned the consequences of suppression for the partners of the regulators. We expected that the partners of participants asked to suppress:

H5. Would display less positive and more negative emotional behavior than would the partners of uninstructed participants;

H6. Would report less positive and more negative emotional experience compared to their counterparts in the no-suppression (control) condition;

H7. Would report diminished feelings of rapport (H7a) and mutual linking (H7b) compared to their counterparts in the no-suppression (control) condition. By contrast, we did not expect to find any effect of suppression on social status (H7c; e.g., English et al., 2013).

H8. Concerning the moderating role of power, we expected that suppression would affect the partners of instructed participants in the equal power condition more than their counterparts assigned to the high-power role, as powerholders are posited to experience higher social distance and to pay less attention to others’ emotional states (Magee & Smith, 2013). Thus, we expected that the partners of suppressors in the equal power condition (cooperators) would show less positive and more negative expressive behavior (H8a) compared to the partners of suppressors in the power disparity condition (managers). Likewise, we predicted that they would report less positive and more negative emotional experience (H8b), as well as diminished feelings of rapport (H8c) and mutual linking (H8d) compared to the partners of suppressors in the power disparity condition (managers).

## Method

### Participants

The sample consisted of 88 participants, *M*_age_ = 23.44, *SD* = 3.63; age range: 20-28; 62.5% females. Italian undergraduate students (in their first, second, and third year of study) constituted 89% of the sample (42% psychology, 7% medicine, 6% political science, 6% foreign languages, 6% media studies, 5% economy, 2% law, 2% architecture, 2% natural sciences, 2% philosophy, 2% engineering, 7% others), while the remaining 11% of participants were employed and had achieved a high school degree or a university bachelor degree. All participants were Caucasian.

The participants were volunteers and received no compensation for their participation in the study.

### Procedure

Approximately half of the undergraduate students were recruited during class lessons (after asking the class professor for cooperation and permission, researchers briefly explained the project and asked students whether they were willing to participate). Other participants were recruited using an announcement on Facebook. Upon giving their consent, participants were asked to provide their email address, which the researchers later used to send them a questionnaire package (including the questionnaires to measure trait suppression and dominance), which they were asked to fill out and send back to the researchers. Participants were randomly paired with a partner of the same gender (for a total of 44 dyads) and individually contacted by email to arrange an appointment to come to the university laboratories.

**Figure 1:**
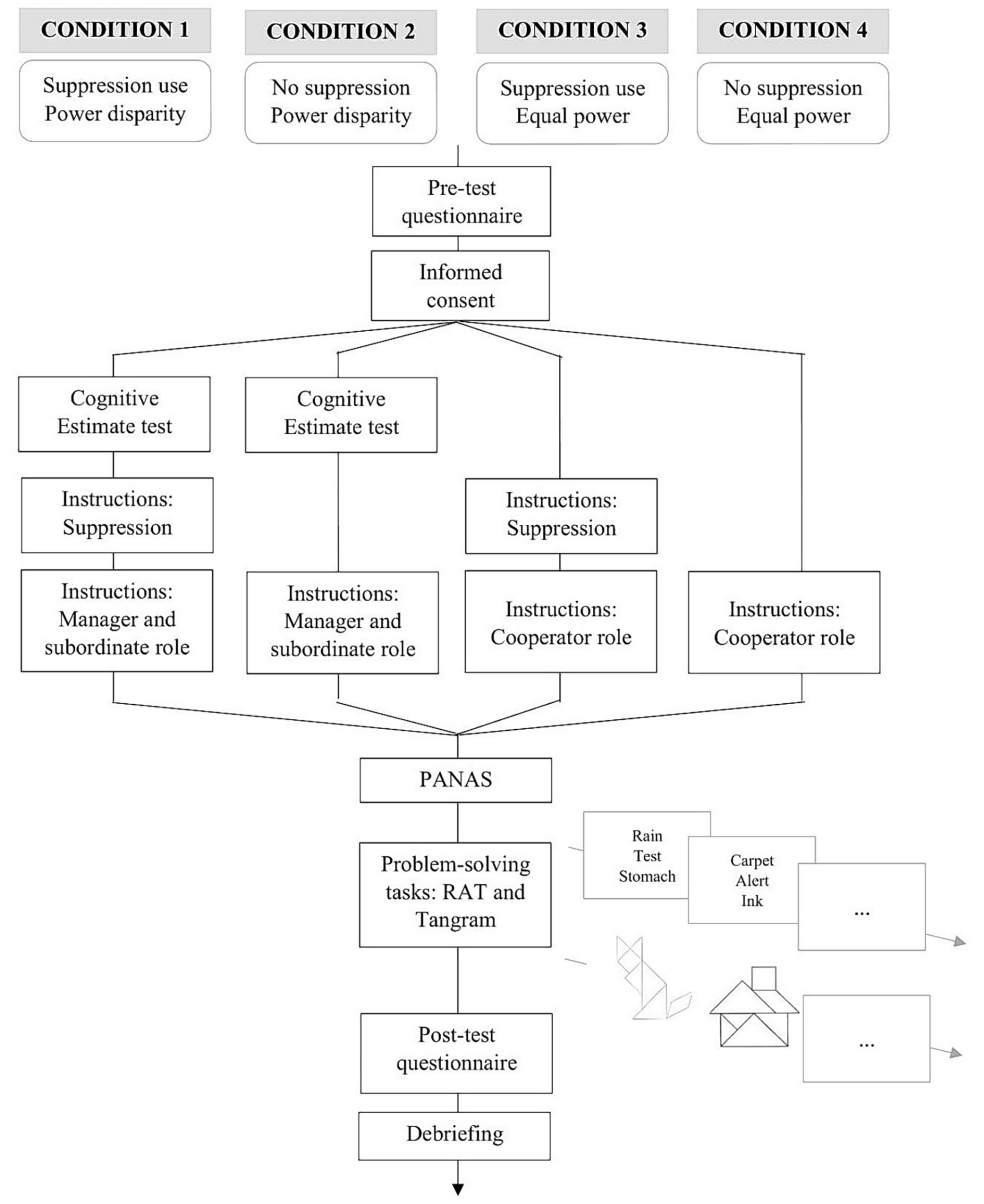
The figure displays the procedure used in the study for each experimental condition to which dyads were randomly assigned.

Dyads were randomly assigned to one of four experimental conditions (see Figure 1). The procedure for manipulating power and role were adapted from Galinsky et al. (2003), while the instructions used to manipulate expressive suppression were adapted from Gross (1998).

#### Condition 1 (Power Disparity, Expressive Suppression)

Unacquainted pairs of participants were introduced to each other (after the experimenter verified that they had not met before). They were seated approximately 1 meter apart on either side of a rectangular desk and were informed that the session would be videotaped. The experimenter explained that the goal of the research was to better understand people’s problem-solving and reasoning abilities and asked the participants to fill out a consent form and to respond to the items of the Cognitive Estimation Test (Della Sala, MacPherson, Phillips, Sacco, & Spinnler, 2003).

After the questionnaires were completed, the experimenter informed the participants that they would be doing two problem-solving tasks and that they would win chocolate candies for each problem they were able to solve. The experimenter explained that the tasks required one person to be the manager and the other to be the subordinate, adding that their responses on the Cognitive Estimation Test would be used to assign one of them to the role of manager. The experimenter then left the room to mark the test answers. In reality, the roles of manager and subordinate were randomly assigned before the participants arrived. After a few minutes, the experimenter came back and asked the participant assigned to be the subordinate to go into a separate room, ostensibly because of a problem with reading his or her handwriting. While in this second room, the subordinate was instructed to use expressive suppression as a strategy to regulate and hide his or her emotional reactions. Specifically, he or she read the following instructions:

Dear collaborator: We ask you not to show your emotional reactions (either positive or negative) when you are working with your partner to solve the two problem tasks. Please try your best not to let your feelings show. In other words, as long as you and your partner are together, please try to behave such that someone watching you would not know that you are feeling anything at all.

After reading these instructions, the experimenter and the participant returned to the previous room and the experimenter announced which of the participants had been selected to be the manager. The manager and the subordinate were given printed instructions about their role and were asked to read them aloud. The ‘manager’ instructions emphasized that he or she would have complete control over the work process and the division of rewards. Specifically, the instructions were as follows:

Dear collaborator: As manager, you are in charge of directing the subordinate in solving the problem tasks. You will decide how to structure the solving process and you will have the last word on the responses to the task questions. If you and the subordinate disagree, you can tell the subordinate, “*Because I am the manager and it’s up to me to make decisions, we will give the answer that I believe is the correct one*”. Your goal is to solve the highest number of problems possible. Moreover, at the end of each task, you are in charge of determining the rewards you and the subordinate will receive. That is, you will decide how the chocolate candies will be divided between you and the subordinate, based on your evaluation of the quality of the subordinate’s work. For instance, you can decide to reward good-quality work or to punish a poor contribution.

The ‘subordinate’ instructions emphasized that he or she would have no control over how the work was done or the divisions of reward. Specifically, the instructions were as follows:

Dear collaborator: As subordinate, you will have the responsibility of carrying out the tasks according to instructions given to you by your manager. You will have to contribute to solving the tasks but keep in mind that the last word will be up to the manager. Your goal is to solve the highest number of problems possible. Moreover, at the end of each task, the manager will be in charge of determining the rewards you will receive. That is, he or she will decide how the chocolate candies will be divided between the two of you, based on his or her evaluation of the quality of your work.

#### Condition 2 (Power Disparity, Free Expression)

In this condition, the procedure was the same as in Condition 1, with the exception that the subordinate was not asked to temporarily leave the room and was not instructed to suppress his or her emotional reactions.

#### Condition 3 (Equal Power, Expressive Suppression)

Before the participants arrived, one of them was randomly assigned to receive the suppression instructions. Upon arrival, they were accompanied in two different rooms and were asked to wait until everything was ready for the session. In the meantime, both participants were asked to read and fill out the consent form. The participant assigned to the suppression condition was also instructed to use expressive suppression as a strategy to regulate and hide his or her emotional reactions using the same instructions as in Condition 1.

Then, both participants were accompanied in the same room and were introduced to each other. The experimenter asked them to collaborate to solve a number of task problems. Specifically, both participants read the following instructions:

Dear collaborator: You are asked to collaborate with your partner to carry out the tasks. As peers, you will decide together how to structure the process and you will have to agree about which is the correct answer to give. You can use sentences like “*I think that this is the correct response, do you agree?*” Your common goal is to solve the highest number of problems as possible. At the end of each task, the chocolate candies you have won will be equally divided between you.

#### Condition 4 (Equal Power, Free Expression)

In this condition, the procedure was the same as in Condition 3, with the exception that neither of the participants was instructed to suppress his or her emotional reactions.

After the participants received the instructions, they were asked to complete the Positive and Negative Affect Schedule (PANAS; Terracciano et al., 2003; Watson, Clark, & Tellegen, 1988) to test possible differences among conditions in the participants’ baseline emotional state. The participants were then given a sheet with the rules of the two problem-solving tasks. In the power disparity conditions, the manager was in charge of reading the rules first and then explaining them to the subordinate; in the equal power conditions, the participants read the instruction sheet together. The first task consisted of an adapted version of the Remote Association Test (RAT; Mednick, 1962). The RAT is a creative, problem solving task measuring people’s ability to connect remote concepts to find a correct solution (Dewhurst, Thorley, Hammond, & Ormerod, 2011; Subramaniam, Kounios, Parrish, & Jung-Beeman, 2009; Taft & Rossiter, 1966). The participants were presented with nine groups of three words each and were asked to find a fourth word semantically connected to them (e.g., light, birthday, church = candle). Participants were given a time of 2 minutes for each group of words. The second task consisted in the Tangram game, in which the players are asked to arrange seven cards of different shapes and sizes so that they match specified templates. The Tangram is a visual, rather than verbal, problem-solving task which has been previously used in experimental research (e.g., Langens & Mörth, 2003; Saleem, Anderson, & Barlett, 2015). In this study, the participants were given three target templates (e.g., a rabbit) and ten minutes to solve each of them. They received three points (and three chocolate candies) for each correct solution. Half of the dyads were asked to solve the RAT first, while the other half were first presented with the Tangram game.

After completing the activities, the participants received their rewards (in Conditions 1 and 2, the manager decided how many candies the subordinate received). They were then asked to individually answer a questionnaire including post-test measures. Finally, they were debriefed about the goals of the study and thanked for their participation.

### Measures

#### Pre-Test Questionnaire

##### Expressive suppression

The four-item scale of the Emotion Regulation Questionnaire (ERQ; Balzarotti, 2019; Balzarotti, John, & Gross, 2010; Gross & John, 2003) was used to assess participants’ habitual use of expressive suppression (e.g., “I control my emotions by not expressing them”). The items are rated on a Likert scale ranging from 1 (*strongly disagree*) to 7 (*strongly agree*). In this study, Cronbach’s alpha was .75.

##### Dominance

Dominance was assessed using the Big Five Adjectives Questionnaire (BFA; Barbaranelli, Steca, & Caprara, 2002). This questionnaire is used to assess the degree to which respondents believe that each of 175 adjectives describes their personality, measured on a Likert scale ranging from 1 (*not at all*) to 7 (*very much*). In this study, we considered only the 10 adjectives (a sum score was computed) referring to the sub-dimension of dominance (α = .81).

##### Pre-test emotional state

The PANAS (Watson, Clark, & Tellegen, 1988) comprises a 10-item Negative Affect (NA) scale (including adjectives such as afraid, distressed, and nervous) and a 10-item Positive Affect (PA) scale (including adjectives such as active, determined, and strong). Respondents rate the extent to which they usually experience each term on a 5-point scale ranging from 1 (*very slightly* or *not at all*) to 5 (*extremely*). The Italian adaptation of the PANAS (Terracciano et al., 2003) has demonstrated robust psychometric properties. In this study, Cronbach’s α were = .84 for PA and α = .82 for NA.

#### Expressive Behavior

Expressive behavior was recorded during each session. Two synchronized JVC video cameras were used, with each camera focused on one participant’s face and upper torso. The videos were scored for each participants’ emotion expressive behavior using an open-source computer software (Virtual Dub) allowing for slow-motion (frame by frame) inspection of the videos, and Microsoft Excel was used to score the presence/absence of behavioral units. A sampling rate of 1 second was used.

The coding scheme included the recording of a selection of the main action units of the Facial Action Coding System (FACS; Ekman, Friesen, & Hager, 2002). Specifically, we considered upper (AU1, AU2, AU4, AU5, AU6, AU7) and lower face action units (AU9, AU10, AU12, AU15, AU17, AU24, AU25, AU26, AU28, AU32; Balzarotti, Piccini, Andreoni, & Ciceri, 2014). One coder who was trained in the use of the coding scheme coded all videotapes, and another certified FACS coder provided reliability ratings on 60 of the 88 recordings (average inter-observer agreement *r* = .79). Both coders were unaware of the experimental condition to which participants were assigned. The coding of a single interaction took approximately 3 hours.

After FACS coding, a dimensional approach was used so that expressive behaviors (i.e., single AUs or combinations) were classified based on their positive or negative valence (Mehu & Scherer, 2015). Each dimension of valence was scored on a three-point scale (from 0 to 2) depending on the intensity of exhibited facial behavior. Reliabilities were good (average inter-observer agreement *r* = .80). Mean scores were then computed for positive and negative expressivity^i^ across the two activities. Analyses were conducted on positive and negative expressivity scores (rather than on AUs raw frequencies).

#### Post-Test Questionnaire: Emotional Experience and Social Outcomes

##### Emotional experience

Following Butler et al. (2003), after the interaction, participants were asked to provide a retrospective report of the emotions that they felt during the interaction. Specifically, participants indicated the extent to which they had felt seven negative emotions (i.e., anxiety, sadness, disappointment, embarrassment, irritation, disgust, fear), and seven positive emotions (i.e., tenderness, amusement, happiness, interest, surprise, enjoyment, pride). The categories were derived from the Geneva Emotion Wheel (Scherer, 2005). Cronbach’s alpha was .70 for the Negative Emotions scale and .80 for the Positive Emotions scale.

##### Subjective authenticity

The respondents were asked to rate the following statements on a 5-point Likert scale: (a) “I felt that I could not express my true self,” (b) “The way I behaved was very different from the way I usually behave,” (c) “I had to become a different person.” Cronbach’s alpha for this composite was α = .81.

##### Rapport

To assess participants’ evaluation of the quality of the interaction, we used two items from Butler et al. (2003): (a) “The interaction was warm and smooth” and (b) “I felt that I ‘clicked’ with my partner.” We also added one ad hoc item (c) “I felt at ease while interacting with my partner.” Cronbach’s alpha for this composite was α = .83.

##### Mutual liking

The participants’ mutual liking and willingness to form a friendship were assessed with the following questions from Butler et al. (2003): (a) “To what extent do you like your partner?” (b) “How well do you think you would get along with your partner in the future?” (c) “To what extent would you be interested in talking to your partner again?” (d) “To what extent is your partner the type of person you could become close friends with?” Agreement with these statements was rated on a scale from 1 (*strongly disagree*) to 5 (*strongly agree*). Cronbach’s alpha for this composite was α = .84.

##### Social status

The participants were asked to assess their partner’s social status using two items adapted from English et al. (2012): (1) “My partner is the kind of person who commands respect” and (2) “My partner is the kind of person who is able to influence others.’’ Agreement with these statements was rated on a scale from 1 (*strongly disagree*) to 5 (*strongly agree*). Pearson correlation was *r* = .57.

### Analytic Strategy

As remarked by Butler et al. (2003), one characteristic of social interaction is that the responses of the two individuals within a conversational dyad may be correlated and violate assumptions of independence. Because of nonindependence, multilevel modelling was run (using SPSS [Version 23]) by means of linear mixed models (LMM; Hoffman & Rovine, 2007) nesting participants (Level 1) within dyads (Level 2). Following Kenny et al. (2006, p. 78) compound symmetry was used to model the covariance between dyad members considering unacquainted pairs of participants as undistinguishable dyads.

Eight models were run, one for each dependent variable. In each model, *Suppression* (yes vs. no) and *Power* (disparity vs. equal) were included as fixed factors at level two. A third dichotomous variable named *Role* was also included in the model as a fixed factor at Level 1 to identify each dyad member (1 = member instructed to be manager/not instructed to suppress; -1 = member instructed to be subordinate/instructed to suppress). The two-way and three-way interactions between the fixed factors were also included in the model. In the equal power, no suppression condition the dyad members were randomly assigned to *Role*. Pseudo-*R*^2^ was computed as a measure of effect size running an empty model (i.e., no predictors except for the intercept) following Kenny et al. (2006, p. 95). Type III tests and parameter estimates of the full models are reported in Supplementary Materials (see Tables S3, S4, S5). Bonferroni adjusted pairwise comparisons were used to analyze significant mean differences (SPSS EMMEANS COMPARE command). Specifically, ‘compare by suppression’ was used to contrast participants asked to use suppression vs. uninstructed participants (H1a, H2a, H3a), as well as partners of participants asked to suppress vs. partners of uninstructed participants (H5, H6, H7). ‘Compare by dyad’ was used to contrast dyad members (H1b, H2b, H3b). Finally, ‘compare by power’ was used to contrast participants in different power conditions (H4, H8).

## Results

### Preliminary Analyses

#### Random Assignment Check

We ran a MANOVA to test differences between experimental conditions on trait variables (*Power* x *Suppression* x *Role*). No significant effects emerged on trait suppression (see Supplementary Materials, Table S1). However, despite random assignment, a significant *Power* x *Role* effect emerged for dominance, *F*(1, 78) = 6.12, *p* = .016, with participants assigned to the role of managers reporting significantly higher dominance than those assigned to the role of subordinates. Thus, grand mean centered trait variables (i.e., trait suppression and dominance) were included as covariates in each model in the main analyses.

#### Baseline Emotional State

No significant differences in baseline emotional state emerged among conditions (see Supplementary Materials, Table S2).

#### Duration of the Interactions

Overall, interactions lasted an average of 29 minutes and 50 seconds (*SD* = 6.05). Participants spent more time on the Tangram game (*M* = 19.31, *SD* = 5.60) than on the RAT (*M* = 10.06, *SD* = 2.04), *F*(1, 40) = 100.43, *p* < .001, η^2^ = .72. Neither suppression, *F*(1,40) = 1.29, *p* = .263, nor power, *F*(1, 40) = 0.63, *p* = .803, influenced overall duration.

### Main Analyses

Descriptive statistics are reported in Table 1.

**Table 1:** Descriptive Statistics: Means and Standard Deviations

Measure	Suppression				Control			
	Power disparity		Equal power		Power disparity		Equal power	
	Manager	Subordinate^a^	Cooperator	Cooperator^a^	Manager	Subordinate	Cooperator	Cooperator
	*M* (*SD*)	*M* (*SD*)	*M* (*SD*)	*M* (*SD*)	*M* (*SD*)	*M* (*SD*)	*M* (*SD*)	*M* (*SD*)
Trait								
Dominance	45.83 (7.95)	38.25 (9.35)	39.50 (7.12)	40.60 (9.83)	46.80 (8.63)	40.60 (9.83)	44.75 (7.49)	45.42 (8.55)
Suppression	2.67 (0.96)	3.25 (1.80)	4.20 (0.90)	3.47 (1.24)	3.19 (0.76)	2.95 (1.32)	3.14 (1.25)	3.01 (1.13)
Nonverbal behavior								
Positive	0.69 (0.35)	0.33 (0.31)	1.56 (0.94)	0.40 (0.27)	1.39 (1.34)	1.64 (0.70)	1.54 (0.78)	1.55 (0.55)
Negative	0.67 (0.45)	0.37 (0.24)	0.72 (0.33)	0.34 (0.15)	0.65 (0.23)	0.95 (0.40)	0.74 (0.36)	0.67(0.26)
Emotional experience								
Positive emotions	2.59 (0.79)	2.61 (0.69)	3.18 (0.74)	2.55 (0.55)	3.42 (0.60)	2.70 (0.33)	3.00 (0.92)	2.94 (0.73)
Negative emotions	1.55 (0.44)	2.23 (0.80)	1.52 (0.50)	1.70 (0.72)	1.50 (0.54)	2.00 (0.65)	1.53 (0.33)	1.61 (0.38)
Social outcomes								
Status	3.58 (1.04)	3.21 (0.62)	3.55 (0.64)	3.30 (0.59)	3.70 (0.54)	3.40 (0.81)	3.46 (0.58)	3.87 (0.56)
Inauthenticity	1.50 (0.64)	3.17 (1.34)	1.63 (0.55)	2.33 (1.27)	1.40 (0.64)	1.40 (0.41)	1.50 (0.50)	1.44 (0.48)
Rapport	3.47 (0.67)	3.36 (0.85)	3.77 (1.01)	3.43 (1.03)	4.17 (0.64)	4.13 (0.74)	3.75 (0.75)	3.87 (0.57)
Mutual liking	3.35 (0.73)	3.25 (0.61)	3.55 (0.44)	3.72 (0.90)	3.45 (0.45)	3.55 (0.66)	3.48 (0.43)	3.38 (0.89)

^a^Participant receiving suppression instructions.

### Positive and Negative Expressive Behavior

The results showed that, consistent with our Hypothesis (H1a), participants instructed to suppress showed less positive behavior than did uninstructed participants both in the power disparity, *M*_diff_ = 1.30, *SE* = 0.30, *p* < .001, 95% CI [0.71, 1.90], and in the equal power condition, *M*_diff_ = 1.13, *SE* = 0.30, *p* < .001, 95% CI [0.54, 1.72]. Likewise, participants instructed to suppress showed less negative behavior than did participants who received no instructions in both the power disparity, *M*_diff_ = 0.58, *SE* = 0.133, *p* < .001, 95% CI [0.31, 0.84], and equal power condition, *M*_diff_ = 0.33, *SE* = 0.133, *p* = .016, 95% CI [0.06, 0.59].

Moreover (H1b), instructed participants showed significantly less positive, *M*_diff_ = 1.22, *SE* = 0.22, *p* < .001, 95% CI [0.77, 1.66], and less negative behavior, *M*_diff_ = 0.40, *SE* = 0.12, *p* = .002, 95% CI [0.16, 0.64], than did their partners in the equal power condition; by contrast, in the power disparity condition, participants instructed to suppress showed less negative expressive behavior than did their partners, *M*_diff_ = 0.30, *SE* = 0.12, *p* = .011, 95% CI [0.07, 0.52], but no difference emerged in positive expressivity, *M*_diff_ = 0.37, *SE* = 0.21, *p* = .085. Thus, this Hypothesis was partially confirmed.

The results also indicated that suppression affected the number of positive, but not of negative expressions displayed by the partners of suppressors in the power disparity condition (H5). Specifically, managers in the suppression condition showed less positive behavior compared to managers interacting with uninstructed subordinates, *M*_diff_ = 0.78, *SE* = 0.30, *p* = .011, 95% CI [0.18, 1.37], while no difference in outward positive expressions emerged between partners of uninstructed and instructed participants in the equal power condition, *M*_diff_ = 0.09, *SE* = 0.30, *p* = .767. Also, the partners of suppressors did not exhibit more negative expressions than did the partners of uninstructed participants in either the power disparity, *M*_diff_ = 0.02, *SE* = 0.14, *p* = .889, or the equal power condition, *M*_diff_ = 0.01, *SE* = 0.15, *p* = .960.

**Figure 2:**
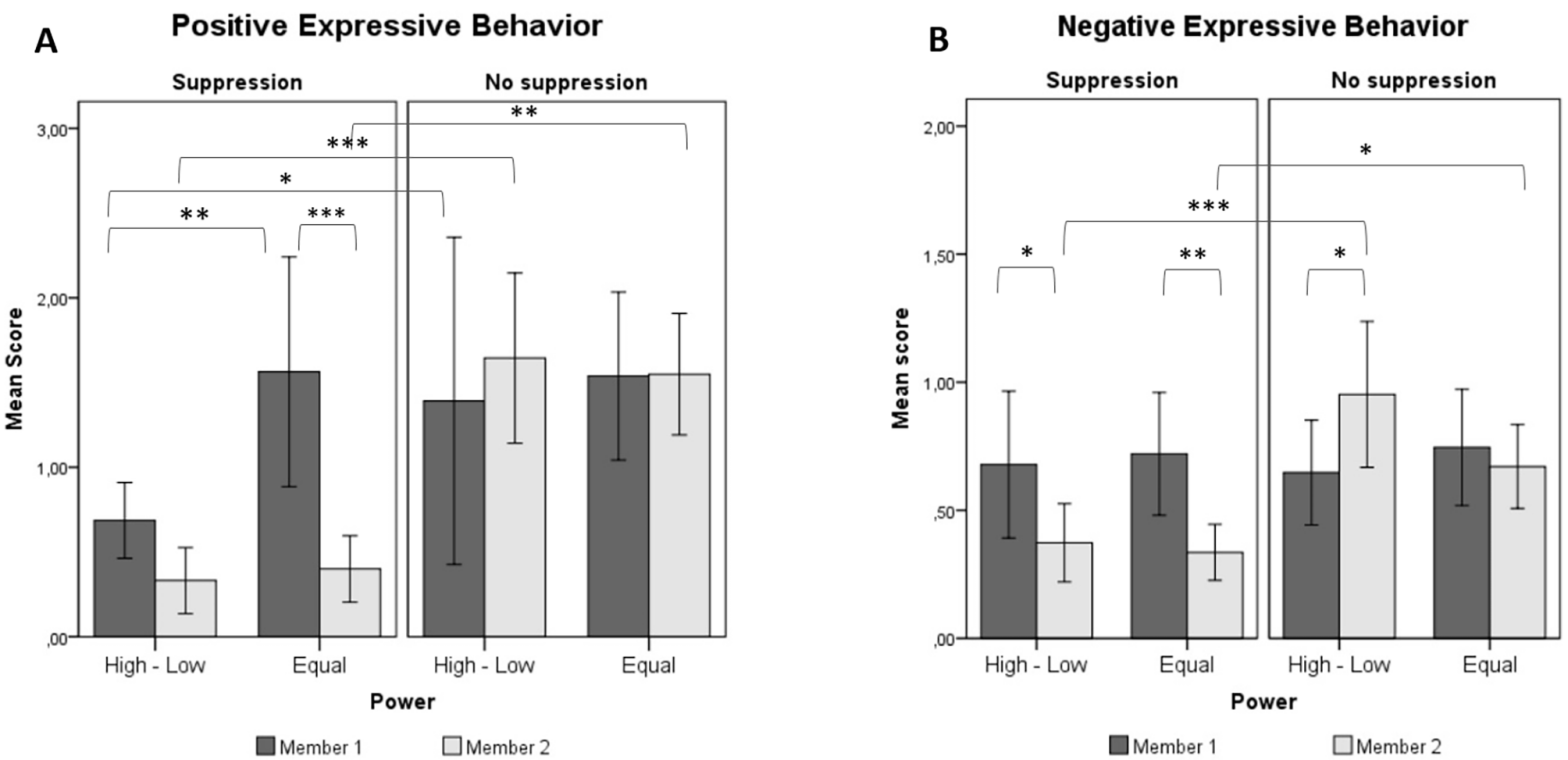
Means and significant differences for A) positive and B) negative nonverbal behavior. Error bars represent 95% confidence intervals. High-Low = power disparity condition; Equal = equal power condition. Member 1 = manager/cooperator; Member 2 = subordinate/cooperator. *p < .05. **p < .01. ***p < .001.

Concerning the moderating role of power (H8a), managers in the suppression condition showed less positive behavior compared to partners of instructed cooperators, *M*_diff_ = 0.99, *SE* = 0.31, *p* = .002, 95% CI [0.37, 1.60]. These results are shown in Figure 2.

### Positive and Negative Emotional Experience

Disgust, fear, and tenderness were excluded from the analyses, as mean scores for these emotions were < 1.5, indicating that most participants rated them as very low.

Concerning the consequences of suppression for the regulator (H2), no significant differences emerged in positive emotional experience between instructed and uninstructed participants (H2a) in either the power disparity, *M*_diff_ = 0.03, *SE* = 0.29, *p* = .907, or the equal power conditions, *M*_diff_ = 0.32, *SE* = 0.29, *p* = .278. Likewise, no difference in negative emotions emerged between instructed and uninstructed participants in either the power disparity, *M*_diff_ = 0.12, *SE* = 0.22, *p* = .414, or the equal power condition, *M*_diff_ = 0.18, *SE* = 0.24, *p* = .442. This Hypothesis was thus not confirmed.

Instructed participants reported lower levels of positive emotions than did their partners (H2b) in the equal power condition, *M*_diff_ = 0.70, *SE* = 0.24, *p* = .006, 95% CI [0.21, 1.19], but not in the power disparity condition, *M*_diff_ = 0.19, *SE* = 0.24, *p* = .447. By contrast, participants instructed to suppress reported a higher rate of negative emotions compared to their partners in the power disparity, *M*_diff_ = 0.61, *SE* = 0.19, *p* = .003, 95% CI [0.23, 1.00], but not in the equal power condition, *M*_diff_ = 0.27, *SE* = 0.21, *p* = .198. Concerning the moderating role of power (H4a), instructed subordinated reported more negative emotions compared to instructed cooperators, *M*_diff_ = 0.55, *SE* = 0.22, *p* = .015, 95% CI [0.11, 1.00], but no significant differences were observed in positive emotions, *M*_diff_ = 0.15, *SE* = 0.30, *p* = .613. These Hypotheses were thus only partially confirmed.

Concerning the consequences of suppression for the partners of suppressors, consistent with our Hypothesis (H6), managers who interacted with instructed subordinates experienced fewer positive emotions than did managers who interacted with uninstructed subordinates, *M*_diff_ = 0.80, *SE* = 0.28, *p* = .005, 95% CI [0.25, 1.35]. However, no significant difference in positive emotions emerged between partners of instructed and uninstructed cooperators, *M*_diff_ = 0.31, *SE* = 0.30, *p* = .310. Likewise, no significant differences in negative emotional experience emerged between the partners of instructed and uninstructed participants in either the power disparity, *M*_diff_ = 0.12, *SE* = 0.22, *p* = .588, or the equal power condition, *M*_diff_ = 0.14, *SE* = 0.22, *p* = .540.

**Figure 3:**
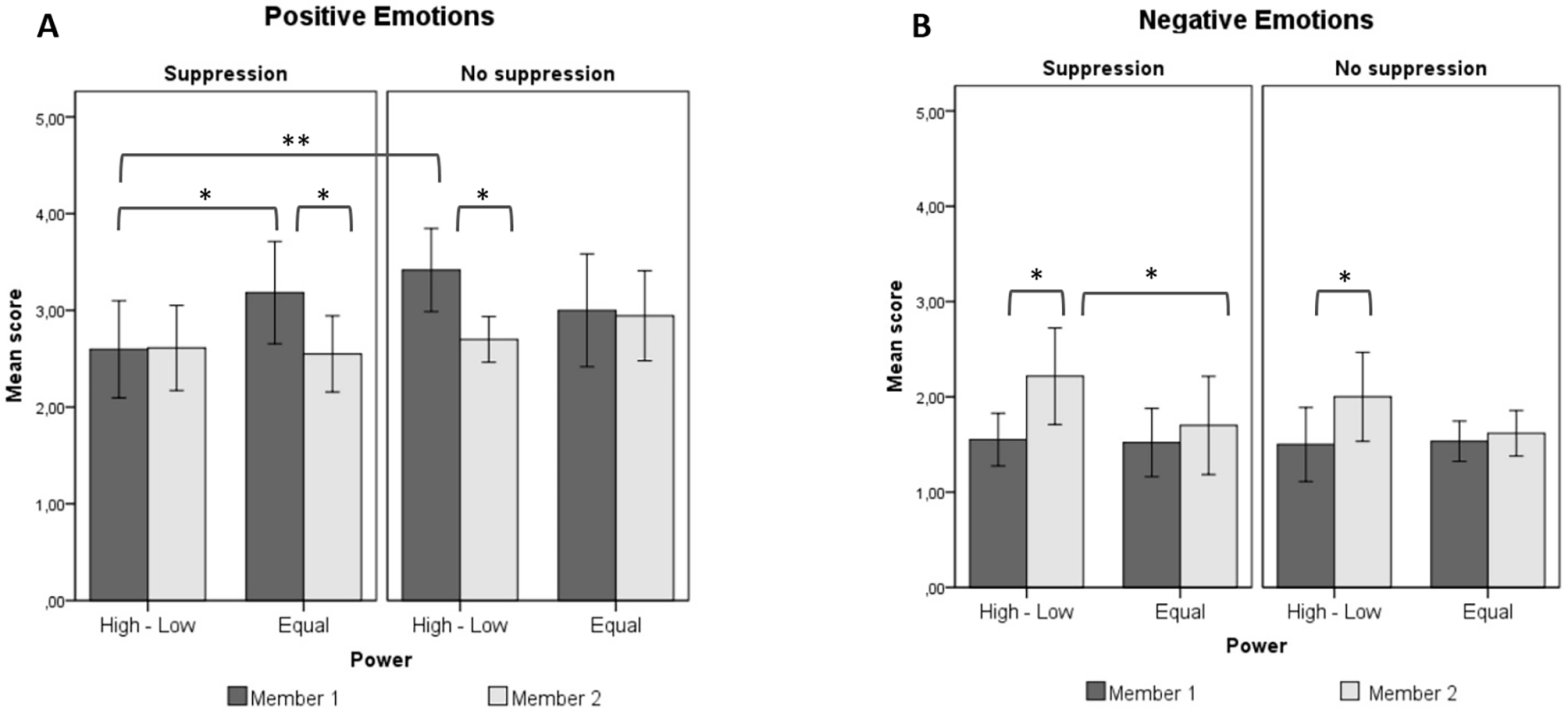
Means and significant differences (linear mixed models) for (A) positive and (B) negative emotional experience. Error bars represent 95% confidence intervals. High-Low = power disparity condition; Equal = equal power condition. Member 1 = manager/cooperator; Member 2 = subordinate/cooperator. *p < .05. **p < .01. ***p < .001.

Concerning the moderating role of power (H8b), partners of instructed cooperators reported higher (rather than lower) positive emotional experience than did managers who interacted with instructed subordinates, *M*_diff_ = 0.74, *SE* = 0.29, *p* = .013, 95% CI [0.16, 1.32]. These results are shown in Figure 3.

### Social Outcomes: Authenticity, Rapport, Mutual Liking, and Social Status

**Figure 4:**
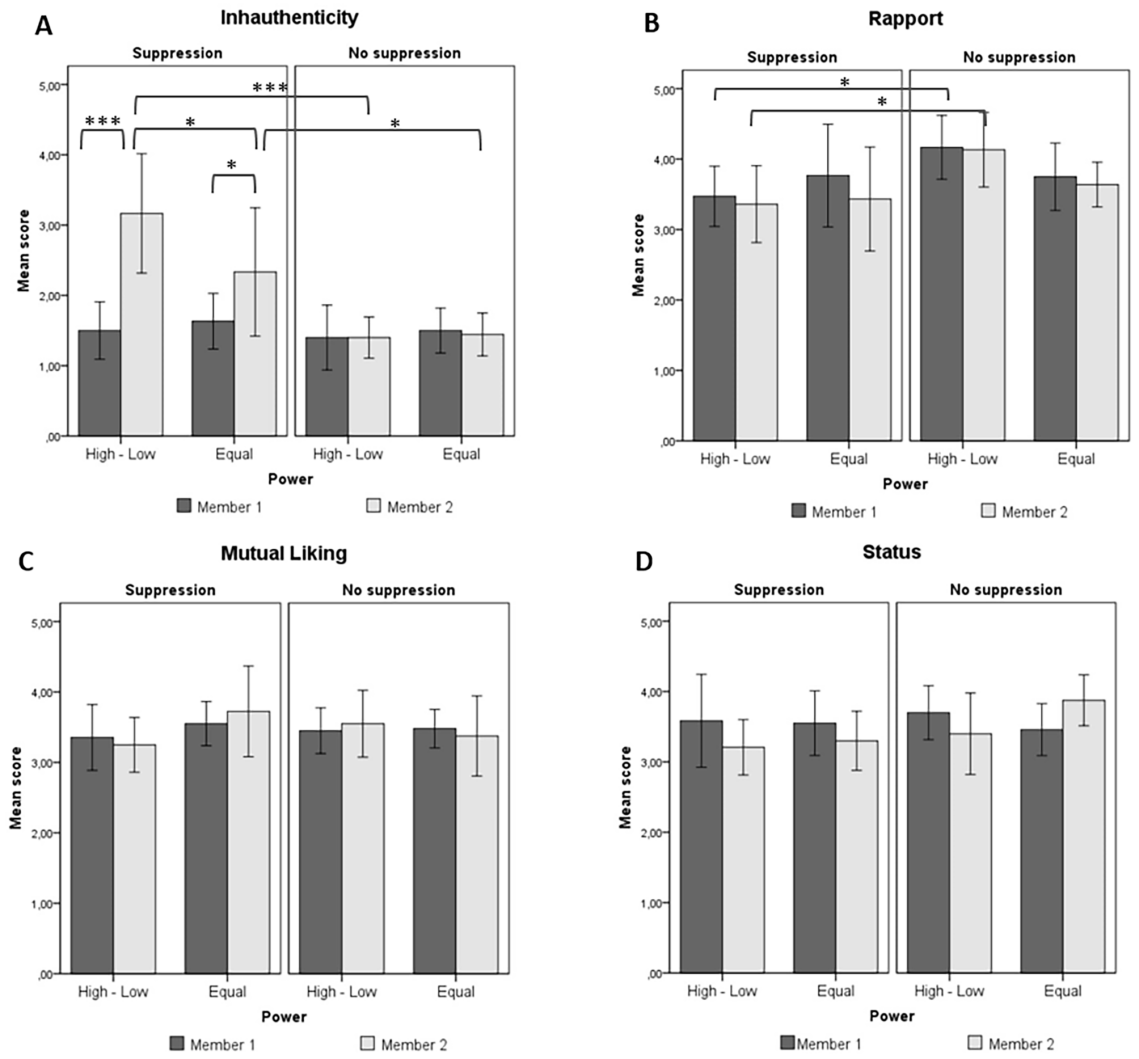
Means and significant differences (linear mixed models) for (A) inauthenticity, (B) feelings of rapport, (C) mutual liking, and (D) social status. Error bars represent 95% confidence intervals. High-Low = power disparity condition; Equal = equal power condition. Member 1 = manager/cooperator: Member 2 = subordinate/cooperator. *p < .05. **p < .01. ***p < .001.

Concerning *subjective authenticity* (H3; Figure 4A), participants who were asked to suppress reported higher inauthenticity than did uninstructed participants (H3a) in both the power disparity, *M*_diff_ = 1.73, *SE* = 0.33, *p* < .001, 95% CI [1.08, 2.38], and equal power conditions, *M*_diff_ = 0.83, *SE* = 0.33, *p* = .013, 95% CI [0.18, 1.48]. Also, instructed participants reported higher inauthenticity than did their partners (H3b) in both the power disparity, *M*_diff_ = 1.58, *SE* = 0.32, *p* < .001, 95% CI [0.98, 2.18], and the equal power conditions, *M*_diff_ = 0.79, *SE* = 0.34, *p* = .017, 95% CI [0.15, 1.43]. Finally, subordinates (H4b) reported higher inauthenticity than did instructed co-operators, *M*_diff_ = 0.85, *SE* = 0.33, *p* = .012, 95% CI [0.20, 1.50].

Concerning *rapport* (Figure 4B), the Hypotheses (H7a) were only partially supported: Partners of suppressors reported lower feelings of rapport than did partners of uninstructed participants in the power disparity condition, *M*_diff_ = 0.68, *SE* = 0.32, *p* = .034, 95% CI [.05, 1.31], but not in the equal power condition, *M*_diff_ = 0.13, *SE* = 0.32, *p* = .696. Moreover (H8c), no significant difference in rapport emerged between managers of instructed subordinates and their counterparts in the equal power condition, *M*_diff_ = 0.43, *SE* = 0.33, *p* = .200. Finally, the results showed that in the power disparity condition instructed participants reported less rapport than did uninstructed participants, *M*_diff_ = 0.72, *SE* = 0.31, *p* = .024, 95% CI [0.10, 1.35].

Concerning *mutual liking* (Figure 4C), no significant effects emerged (see Supplementary Materials, Table S5). The Hypotheses (H7b, H8d) were thus not confirmed.

Finally, concerning *status* (H7c; Figure 4D), the analysis yielded no significant effects, with the exception of trait dominance, *Β* = 0.02, *SE* = 0.009, *t* = 2.38, *p* = .019, 95% CI [0.01, 0.04]. The participants reporting higher dominance tended to be perceived by their partners as more able to gain the respect of and influence over others.

## Discussion

One common way to regulate one’s emotions is to hold back from showing them. The present study adds to the existing literature investigating how using this regulatory strategy − called expressive suppression − impacts the individuals’ social functioning (e.g., Butler et al., 2003, 2007; English et al., 2012). Specifically, while previous studies have mostly investigated the consequences of suppression within the context of close relationships, we examined the interpersonal outcomes of instructed suppression within task-oriented dyads (Gross et al., 2004), testing whether power differences moderate these effects. Temporary task-oriented dyads (or groups) represent a type of social context in which unacquainted individuals are assigned to work together on a task (Meyerson et al., 1996), which requires them to engage in cooperative and coordinated activity.

Concerning the personal consequences of suppression for the regulators (H1-H4), consistent with our Hypothesis (H1a), the results showed that the participants asked to suppress exhibited less positive and less negative emotional behavior than did uninstructed participants in both the power disparity and the equal power conditions, thus indicating that the instructions were effective. Moreover, consistent with the Hypothesis of reduced expressivity (H1b), instructed participants exhibited fewer positive and negative expressions than did their partners, even though the difference in positive expressivity between the two members of the dyad was significant in the equal power condition only. Thus, the participants assigned to the low-power role and asked to suppress their emotional reactions showed levels of positive emotional behavior similar to those of their partners assigned to the high-power role. This result seems to indicate that, in the power disparity condition, the use of suppression reduced the number of positive expressions displayed by the regulator as well as by his/her partner.

When looking at self-reported emotional experience, our Hypotheses (H2) were overall not confirmed. First (H2a), we found no significant differences between instructed and uninstructed participants in terms of self-reported (positive and negative) emotional experience in either the power disparity or the equal power condition; thus, the study failed to replicate the negative impact of suppression on the regulator’s self-reported emotions. This result may be due to the fact that we addressed positive and negative emotions generated by the situation in general (including the emotions elicited by the tasks) rather than specifically asking participants to report the emotions they experienced in response to the interaction with their partner. Previous studies (see Butler et al., 2003) have also found a significant negative influence of suppression on regulators’ emotional experience, but this influence is limited to the emotions provoked by the (disrupted) interaction with one’s partner. Second (H2b), we found that instructed participants reported less positive and more negative emotional experience than did their partners, but this difference was limited to the equal power condition for positive emotions and to the power disparity condition for negative emotions. In other words, participants asked to suppress and to cooperate as peers reported less positive emotional experience than did their partners, while participants asked to suppress and assigned to the low-power role reported more negative emotional experience than did their partners assigned to the high-power role.

Concerning social outcomes, our Hypotheses about inauthenticity were confirmed (H3): Overall, regulators reported feeling more inauthentic than did uninstructed participants in both high/low and equal power conditions. This finding extends previous evidence regarding the association of expressive suppression with diminished perceived authenticity in close relationships (English & John, 2013; Gross & John, 2003) to the social context of temporary task-oriented dyads. In addition, the results indicated that regulators reported less feelings of rapport than did uninstructed participants in the power disparity condition.

Finally, our Hypothesis concerning the moderating role of power (H4) was only partially confirmed. Specifically, we found that instructed participants in the power disparity condition reported higher inauthenticity and more negative emotions than did instructed participants in the equal power condition, thus indicating more detrimental consequences of suppression for the participants assigned to the low-power role. However, no difference emerged in positive emotional experience between the regulators in the power disparity and equal power conditions.

Concerning the consequences of suppression for the partners of the regulators (H5-H8), our Hypotheses were only partially confirmed. We found that (H5) suppression affected the display of positive emotional behavior of the partners of the regulators in the power disparity condition: Managers interacting with instructed subordinates exhibited a lower number of positive expressions than did managers interacting with uninstructed subordinates. Moreover, the results also showed that the participants assigned to the high-power role reported lower levels of positive emotional experience (H6) as well as lower feelings of rapport (H7a) than did participants assigned to the high-power role interacting with an uninstructed subordinate. However, no significant differences emerged in either negative emotional behavior or negative emotional experience. Finally, unlike participants assigned to the role of manager, partners of instructed participants in the equal power condition did not report less positive and more negative affect or less rapport than did partners of uninstructed participants.

Concerning the Hypothesis about the moderating role of power (H8), we found differences between the partners of the regulators in the power disparity condition and the partners of instructed participants in the equal power condition, but not in the expected direction. Instead, the participants assigned to the manager role exhibited significantly *fewer* positive expressions and reported *less* positive emotional experience than did the partners of suppressors in the equal power condition. Thus, although previous literature suggests that powerholders experience higher social distance (Magee & Smith, 2013) and pay less attention to others (Fiske, 1993; Goodwin et al., 1998; Keltner & Robinson, 1996, 1997), in our results managers showed less positive emotional behavior when their subordinates were induced to hide their feelings and reported lower positive emotional experience than partners of instructed cooperators. One possible explanation may be related to violated role expectations (Hall et al., 2001): It has been argued that high-power individuals expect low-power individuals to make explicit efforts to affiliate with them, because of the latter’s dependence (Magee & Smith, 2013). When subordinates are induced to use suppression, however, their reduced expressive behavior may be interpreted by managers as withdrawal and thus as role-incongruent. Also, powerholders are motivated to judge their subordinates (our instructions explicitly asked managers to judge their partners’ performance), and this may lead them to be more sensitive to expectancy-disconfirming behaviors (Hall et al., 2001).

Overall, our findings seem to suggest that the use of suppression to regulate one’s emotions may negatively influence task-oriented interactions between individuals assigned to high and low power positions more than interactions between individuals sharing equal power. Although the existing literature has so far suggested that the use of suppression impairs the formation and maintenance of close relationships (e.g., Butler et al., 2003; Srivastava et al., 2009), our results seem to indicate that suppression may also lead to detrimental consequences for the individual’s social functioning within the context of task-focused dyads − that is, within social contexts that are not characterized by intimacy and closeness. In more detail, we found that the use of suppression as a regulatory strategy may have negative implications for interactions involving social distance: When the low-power participants had to hide their emotional reactions, this led the regulators to report heightened negative emotions, inauthenticity, and lower feelings of rapport. The high-power participants were also affected: Participants assigned to the role of manager and asked to interact with a suppressor showed less positive behavior and reported lower positive emotional experience, as well as lower feelings of rapport than did participants assigned to the high-power role interacting with an uninstructed subordinate.

In our results, two social outcomes were not affected by the use of suppression as regulatory strategy: Sociometric standing and mutual liking. The judgments about one’s ability to influence other people (social status) were influenced by trait dominance only. This result is consistent with prior literature (English et al., 2013; Gross & John, 2003; Srivastava et al., 2009), which has found no significant effects of suppression on this social outcome. Finally, although we found that participants assigned to the roles of subordinates and managers reported lower feelings of rapport when subordinates were experimentally induced to hide their feelings rather than free to express them, no significant effects emerged concerning participants’ reports of mutual liking and willingness to form a friendship. This social aspect was not affected by either suppression or power. One possible explanation for this may be related to the type of social context considered in this study: In temporary task-oriented dyads the relational dimension is minimized (Gross et al., 2004), as individuals have never worked together and do not expect to work together again. Moreover, the task-oriented nature of the interaction does not entail any form of emotional sharing or disclosure.

### Limitations and Future Directions

Although the results of the current study are promising, several limitations should be noted. The first important limitation is the relatively small sample size (Du & Wang, 2016), which reduced statistical power, making the analyses less able to detect an effect of a small size and increasing the likelihood of incorrectly accepting the null Hypothesis. The small sample size may also explain our partial failure in the random assignment of participants to the experimental conditions, as the group of individuals assigned to the role of manager reported higher trait dominance. Both trait personality measures, however, were included in the analyses as covariates and the analyses yielded few significant effects. Our results, however, should be interpreted with caution and need further replication by future research.

Second, the use of an experimental paradigm (i.e., instructions, laboratory setting) may limit the generalizability of our results to everyday life. Although we believe that the experimental design also represents a strength of the current study, as it allows us to infer causality, further research is needed to further examine the interaction between the use of suppression as a strategy to regulate emotions and individuals’ power differences. For instance, future research could examine the consequences of suppression within dyadic or group social contexts in which individuals share a task (e.g., collective performance, goals) besides social concerns (e.g., relationships within the group, cohesion; Carron & Brawley, 2000) and have more or less uneven power roles (e.g., work groups, sport teams, musical groups; Gaggioli et al. 2017; Wagstaff, Hanton, & Fletcher, 2013).

A third limitation is our focus on same-sex dyads. Although we included both male and female dyads, future work could examine the personal and social consequences of suppression in the context of mixed-sex dyads or groups. Also, for the purpose of simplifying our design, subordinates but not managers received instructions to suppress. This choice was based on the fact that most literature examining emotional expression within relationships characterized by power differences (leaders-subordinates) has so far focused on the influence that leader’s emotional displays can exert on subordinate’s mood, motivation, and performance (e.g., Sy, Côté, & Saavedra, 2005; Tjosvold, 1984; Van Kleef et al., 2009), while comparatively little is known about whether subordinate’s emotional displays may affect leader’s mood and behavior.

### Conclusions

Despite the aforementioned limitations, our research provides important insight about the social consequences of expressive suppression. Overall, our findings seem to suggest that – within the social context of task-oriented dyads ‒ suppression may impair task-focused interactions between people with high-low power more than interactions between people sharing equal power. Individuals assigned to a low-power role (subordinates) and using suppression to regulate their emotions reported more negative emotional experience than did individuals assigned to equal-power roles, as well as more inauthenticity and diminished feelings of rapport compared to low-power individuals who were free to express their feelings. Notably, we found that the use of suppression also influenced their high-power partners (the individuals assigned to the role of manager), who exhibited less positive behavior, and reported less positive experience and lower feelings of rapport. By contrast, suppression had few effects when individuals were assigned to equal power roles: We found that participants asked to suppress in the equal power condition reported less positive experience and heightened feelings of inauthenticity.

In sum, these results provide initial promising evidence that ‒ although suppression may be less detrimental in non-close social contexts such as task-oriented dyads than in close relationships, power differences could play a role in moderating its personal and interpersonal negative effects. This role deserves further attention in future research.

## Notes

For this article the following supplementary materials are available (Balzarotti, Cesana, Biassoni, & Ciceri, 2020):

- Tables S1 and S2 report the results of preliminary analyses.
- Tables S3-S5 display the results of Linear Mixed Models (Type III Tests and Parameter Estimates) for all the dependent variables included in the study.
